# Course of maternal fatigue and its associated factors during the first 6 months postpartum: a prospective cohort study

**DOI:** 10.1002/nop2.130

**Published:** 2018-02-21

**Authors:** Hiroko Iwata, Emi Mori, Akiko Sakajo, Kyoko Aoki, Kunie Maehara, Koji Tamakoshi

**Affiliations:** ^1^ Graduate School of Nursing Chiba University Chiba Japan; ^2^ Graduate School of Medicine Nagoya University Aichi Japan

**Keywords:** fatigue, midwifery, multivariate, nurses, nursing, parent, postpartum

## Abstract

**Aims:**

To identify the course of maternal fatigue during the first 6 months postpartum and to determine factors associated with it.

**Design:**

A prospective cohort study.

**Methods:**

Women (*N *=* *2,697) in 13 Japanese hospitals provided longitudinal data using self‐report questionnaires at five time points. Maternal fatigue was assessed using the Postnatal Accumulated Fatigue Scale. We focused on the effect of maternal age and parity on the course of maternal fatigue and used a mixed between/within‐subjects analysis of variance. Factors associated with maternal fatigue were analysed using stepwise multiple regression.

**Results:**

In the 6‐month postpartum period, the level of fatigue was highest at 1 month and significantly decreased from 1–4 months postpartum. Primiparas showed a significantly higher level of fatigue than multiparas during hospital stay and their levels of fatigue more closely approximated the 1‐month peak. Multiparas showed significantly higher levels of fatigue than younger primiparas at 6‐month postpartum. Factors associated with maternal fatigue included satisfaction with sleep, concerns about child‐rearing, satisfaction with social support, financial burden and meal times per day.

## 1 INTRODUCTION

Many new mothers report fatigue after childbirth. In Japan, up to 67% of mothers reported tiredness or fatigue at 1 month postpartum (Shimada et al., 2006). Care giving to infant requires both physical and mental effort. Infants’ irregular sleep patterns and frequent feeds are typical in the early postpartum period and may contribute to maternal fatigue.

Fatigue has been reported to cause unfavourable effects on maternal well‐being, daily functioning and mother‐infant interaction. For example, fatigue diminishes mothers’ ability to concentrate or tolerate children and increases parenting stress, which damages their daily functioning, communication with others and mother‐infant relationships (Giallo, Gartland, Woolhouse, & Brown, 2016; Giallo, Rose, Cooklin, & McCormack, 2013; Kurth, Kennedy, Spichiger, Hösli, & Stutz, 2011). However, maternal fatigue is often ignored as an expected part of parenting and priority placed on breastfeeding and mother–infant interaction.

## 2 LITERATURE REVIEW

### 2.1 Concept of maternal fatigue

Postpartum maternal fatigue can be conceptualized as a multidimensional concept with physical, emotional (Milligan, Lenz, Parks, Pugh, & Kitzman, 1996) and cognitive (Tsuchiya et al., 2016) aspects. Physical aspects of fatigue include feelings of tiredness or exhaustion. Emotional aspects may involve feelings of anxiousness or depression, while cognitive aspects may include feeling unfocused or unmotivated (Tsuchiya et al., 2016). Fatigue and tiredness are terms often used synonymously. However, fatigue is “more severe and prolonged, is a negative feeling and is not as easily relieved” compared with tiredness (Milligan et al., 1996, p;.288). Although there is partial overlap in the meaning of fatigue and depression, they have been understood as separate concepts in nursing academia (Giallo, Gartland, Woolhouse, & Brown, 2015; Kuo, Yang, Kuo, Tseng, & Tzeng, 2012; Milligan et al., 1996). In this study, we define maternal fatigue as “subjective experiences of the whole body, encompassing the physical, emotional and cognitive functioning” in postpartum mothers (Tsuchiya et al., 2016, p.6).

### 2.2 Prevalence of maternal fatigue

Prevalence of reported maternal fatigue varies between countries. Available data show that 67% of mothers reported fatigue at 1 month postpartum in Japan (Shimada et al., 2006), 82% at 3 months postpartum in South Korea (Song, Chae, & Kim, 2014), 69% at 6 to 7 months postpartum in Australia (Brown & Lumley, 2000) and 18% at 1 year postpartum in the Netherlands (Bakker, van der Beek, Hendriksen, Bruinvels, & van Poppel, 2014). These data provide a point prevalence but cannot be compared because of different measurement times and tools.

Information regarding the course of maternal fatigue in the postpartum period is still limited. Kuo et al. (2012) examined 121 mothers in Taiwan from the third trimester to 1 week postpartum and found that the level of fatigue was highest at 1 day postpartum, showing a decrease towards the seventh postpartum day. Another study conducted with 197 mothers in Taiwan from the second trimester to 1 month postpartum reported that the level of fatigue increased from the second to third trimester and remained unchanged until 1 month postpartum (Cheng, Chou, Wang, Tsai, & Liou, 2015). In Australia, Giallo, Seymour et al. (2015) examined 70 mothers from three to 7 months postpartum and found that their levels of fatigue remained unchanged over time. A limitation of these longitudinal studies is that the findings are based on small sample sizes with a limited postpartum period. Larger scale longitudinal studies will be needed for comprehensive understanding of the course of maternal fatigue.

### 2.3 Factors associated with maternal fatigue

Previous studies have identified several factors associated with maternal fatigue. Poor sleep quality was associated with severer fatigue at 1 month (Cheng et al., 2015) and three to 7 months postpartum (Giallo, Seymour et al., 2015). Perceived social support at 1 month postpartum (Cheng et al., 2015) and the amount of infant crying during the first 3 months (Kurth et al., 2011) were also associated with maternal fatigue. Some demographic/clinical characteristics associated with higher levels of maternal fatigue are: lower socio‐economic status (Giallo, Seymour et al., 2015), caesarean delivery (Lai, Hung, Stocker, Chan, & Liu, 2015) and primiparity (Hattori & Nakajima, 2000).

In studying postpartum fatigue, Pugh and Milligan (1993) provided a useful framework consisting of four factors that influence fatigue: situational factors (e.g. demographics, lifestyle); physiological factors (e.g. clinical characteristics); performance factors (e.g. childcare activities) and psychological factors (e.g. anxiety). Considering extant findings of this framework, studies that investigated performance or psychological factors regarding maternal fatigue have been scarce. Furthermore, most studies to date have investigated factors associated with maternal fatigue at a specific time point, failing to identify factors that influence longer term fatigue during the postpartum period.

### 2.4 Significance of this study

Research to date has identified the course of maternal fatigue and its associated factors during the postpartum period. However, findings of those studies were mostly based on cross‐sectional studies using a small sample size with a limited postpartum period. A large‐scale longitudinal study will serve to better understand the course of maternal fatigue and its associated factors during a longer term postpartum period.

This study had two aims: 1) To describe the course of maternal fatigue during the first 6 months postpartum; and 2) To determine associated factors during the same time period. In particular, we sought to assess the effect of maternal age and parity for the first aim because this has been our primary concern throughout our research project (Mori, 2014). For the second aim, we sought to explore factors associated with maternal fatigue in two times: from hospital stay to 1 month postpartum and from 1–6 months postpartum.

## 3 METHODS

### 3.1 Study design and participants

We undertook a multicenter prospective cohort study in two regions of Japan: Kansai and Kanto, both densely populated urban areas. The cohort study was based in Japan and was originally designed as one of three studies in the research project: “Developing nursing guidelines for child‐rearing support in older Japanese primiparas” (Mori, 2014). Maternal fatigue was one of five main outcome measures in this larger research project. Findings related to the other main outcomes have been reported elsewhere, viz. depression (Third‐person, Mori, Tsuchiya, Sakajo, Maehara et al., 2015; Third‐person, Mori, Sakajo, Aoki et al., 2016; Third‐person, Mori, Tsuchiya et al., 2016); physical symptoms, breastfeeding (Sakajo et al., 2014) and maternal role confidence and satisfaction (Maehara, Mori, Tsuchiya, Iwata, Sakajo, Ozawa et al., 2016; Maehara, Mori, Tsuchiya, Iwata, Sakajo & Tamakoshi 2016). We recruited participants from 13 hospitals between May 2012 and September 2013. Eligible participants were Japanese women aged 16 years and over, between 0 and 4 days postpartum, delivered a live singleton vaginally or operatively and had a fixed address during the 6 months after recruitment. Exclusion criteria were women with difficulty communicating in Japanese and those with serious health problems in either the mother or newborn. We defined serious health problems as any life‐threatening or distressing disease, such as cancer, severe mental illness and congenital disorders. Researchers or research nurses approached potential participants during their hospital stay following childbirth and explained the study using a brochure. We obtained written informed consent from all participants. The study was approved by the appropriate institutional review board and all relevant institutional committees of participating hospitals. All procedures performed in this study were in accordance with the ethical standards laid down in the 1964 Declaration of Helsinki and its later amendments or comparable ethical standards.

### 3.2 Sample size

We calculated the sample size for the present cohort study based on one main outcome, postpartum depression. We assumed a 20% prevalence of postpartum depression and an expected loss to follow‐up of 40% during the 6 months postpartum. We estimated that approximately 5,000 mothers were needed with 80% power and 5% significance (Third‐person, Mori, Tsuchiya, Sakajo, Maehara et al., 2015).

### 3.3 Measures

We collected data using self‐report questionnaires administered at five times: during hospital stay after childbirth; 1 month postpartum; 2 months postpartum; 4 months postpartum and 6 months postpartum. In addition, data from medical records were collected by the researcher or research nurses. We telephoned all non‐responders. The questionnaires included the following: maternal fatigue, situational factors, physiological factors, performance factors and psychological factors (Pugh et al. 1993).

#### 3.3.1 Maternal fatigue

The Postnatal Accumulated Fatigue Scale (PAFS) (Tsuchiya et al., 2016) was used to assess maternal fatigue and included questions covering the three areas of physical, emotional and cognitive fatigue. The PAFS comprises 13 items with each coded on a three‐point response rating where 0 = rarely, 1 = sometimes and 3 = often. Total scores range from 0 to 39 with higher scores indicating a higher level of fatigue. Examples of items in each of the three domains included “I feel tired in the morning” (physical), “I feel depressed” (emotional) and “I feel unfocused” (cognitive). The PAFS has acceptable convergent and divergent validity and good internal consistency (Tsuchiya et al., 2016). In this study sample, Cronbach's alpha values ranged from 0.86 to 0.89 for the five times.

#### 3.3.2 Situational factors

We obtained the following demographic data by 1 month postpartum: maternal age, education, marital status, employment and financial burden. Data regarding daily maternal activities included total sleep time the previous night and meal times per day, all of which were obtained for all five times.

#### 3.3.3 Physiological factors

The following data were obtained from medical records to provide details of participants’ clinical characteristics: type of delivery, parity, gestational weeks, hospitalization during pregnancy, pregnancy complications and postpartum anaemia. We also obtained data regarding infants’ clinical characteristics: birth weight and long‐term complications from medical records. Feeding methods were evaluated by a single questionnaire item (breastfeeding vs. mixed feeding vs. formula) for each of the five times.

#### 3.3.4 Performance factors

Data regarding childcare activities (e.g. frequency of feeding during the night, duration between feeding and infant bedtime) were obtained by a single questionnaire item with open‐ended responses at each of the five times.

#### 3.3.5 Psychological factors

For this study, we chose three psychological factors: satisfaction with childbirth, concerns about child‐rearing and satisfaction with social support. We did not choose depression because it has been reported as an overlapping concept with fatigue (Giallo, Gartland et al., 2015; Kuo et al., 2012; Milligan et al., 1996) and that depression should not be used to determine unique factors associated with maternal fatigue.

Satisfaction with childbirth was evaluated by the single question, administered during the hospital stay after childbirth: “How do you feel about your childbirth this time?” using a 4‐point scale response (1 = very satisfied, 2 = a little satisfied, 3 = a little dissatisfied, 4 = very dissatisfied). Satisfaction with sleep the previous night was evaluated by the question: “How do you perceive your night's sleep last night?” using a 4‐point scale response (1 = very sufficient, 2 = sufficient, 3 = insufficient, 4 = very insufficient) at each of the five times.

Concerns about child‐rearing were evaluated using 11 items developed by the authors for this study. We assume this measure has acceptable internal consistency and construct validity with three factors explaining 42.3% of the variance: concerns about newborn caretaking (Cronbach's alpha = 0.71); concerns about one's own life (Cronbach's alpha = 0.69); and concerns about social support (Cronbach's alpha = 0.62) (Third‐person, Mori, Tsuchiya, Sakajo, Maehara et al., 2015). Examples of items focusing on each of these concerns included: “I don't have sufficient knowledge about my baby and baby's health”, “I'm afraid I'm not strong enough to take care of my baby” and “I think I can obtain sufficient support for my housework and childcare.” Each item was coded using a 4‐point rating scale where 1 = disagree, 2 = disagree a little, 3 = agree a little, 4 = agree. Total scores range from 11–44 with higher scores indicating more concerns about childcare and daily life. In this sample, Cronbach's alpha values were 0.80 during hospital stay after childbirth and 0.81 at 1 month postpartum. We did not use this measure at 2, 4 and 6 months postpartum.

Satisfaction with social support was assessed using four items developed by the authors for this study. Participants were asked to what extent they are satisfied with four types of social support: instrumental (support with childcare and housework); informational (support with childcare information); appraisal (support that made the participant feel respected or admired) and emotional support (opportunity to share complaints or worries). Each type of social support was evaluated by a single question. For example: “S/he helped me with childcare and housework” for instrumental support; “S/he helped me by providing useful information” for informational support; “S/he acknowledged my effort and praised me” for appraisal support; and “S/he shared my complaints and worries” for emotional support. Every item was coded using a 4‐point response rating where 1 = very satisfied, 2 = a little satisfied, 3 = a little dissatisfied, 4 = very dissatisfied at one, 2, 4 and 6 months postpartum. This measure was not used during hospital stay.

### 3.4 Statistical analysis

Descriptive statistics of means and standard deviations were calculated for continuous variables (e.g. age) and percentages were calculated for categorical variables (e.g. type of delivery). Responses to satisfaction with childbirth were dichotomized as “1 = very satisfied/a little satisfied or 2 = a little dissatisfied/very dissatisfied”. Responses to satisfaction with sleep the previous night were dichotomized as “1 = very sufficient/sufficient/insufficient or 2 = very insufficient”. Responses to satisfaction with social support were dichotomized as “1 = very satisfied/a little satisfied or 2 = a little dissatisfied/very dissatisfied”. The chi‐square test was performed to compare group differences on variables relating to participant demographics and background information.

For our first aim, to identify the course of maternal fatigue during the first 6 months postpartum, focusing on the effect of maternal age and parity, we divided participants into four groups: younger primiparas (aged <35 years), older primiparas (aged ≥35 years), younger multiparas (aged <35 years) and older multiparas (aged ≥35 years). We conducted a mixed between/within‐subjects analysis of variance to assess the impact of group type on mean PAFS scores across the five time periods. We also performed post hoc tests using the Bonferroni correction.

For our second aim, to determine factors associated with maternal fatigue, we used stepwise multiple regression analysis. We performed two sets of analyses in two time periods: from hospital stay to 1 month postpartum and from 1–6 months postpartum. The reason for dividing the 6 month period into these two time periods was that the course of maternal fatigue (i.e. analysis of our first study aim) indicated a change in course of maternal fatigue at 1 month postpartum.

The dependent variables for each analysis were: the total PAFS score at 1 month postpartum minus the total PAFS score during hospital stay and the total PAFS score at 6 months postpartum minus the total PAFS score at 1 month postpartum. Adjustment was done for the initial level of maternal fatigue for each analysis at two times. That is, the total PAFS score during hospital stay was used as a control variable for the first time (from hospital stay to 1 month postpartum) and the total PAFS score at 1 month postpartum was used as a control variable for the second period (from 1–6 months postpartum). We also used maternal age, type of delivery and parity as control variables. Explanatory variables were determined by performing univariate analyses and those found to be significant (*p *<* *.05) were entered into stepwise multiple regression analysis. Predictors reported in previous studies were also used as explanatory variables. We assessed multicollinearity between the explanatory variables. We used SPSS version 21 (SPSS, Armonk, New York, USA) for all analyses.

## 4 RESULTS

Of 3,769 Japanese women who agreed to participate, 2,778 women (73.7% response rate) remained until 6 months postpartum. After exclusion of 81 cases (19 who did not respond within the requested timeframe, 19 with a previous history of a mental disorder and 43 with missing values on the PAFS), data from 2,697 women were available for analysis.

### 4.1 Sample characteristics

Table 1 shows demographic and background information for the 2,697 participants. The mean maternal age was 33.0 years. Most (98.0%) were married, 53.6% were not employed, 54.8% had graduated from junior/high/vocational school or junior college and 83.1% delivered vaginally. Many group differences were observed: older primiparas were more likely to be single, employed, have the least financial burden and have the highest likelihood of caesarean section, whereas younger multiparas were more likely to have a lower education level (all *ps* <.001).

**Table 1:** Participants’ demographic and background information by group type

Variable	Total(*N *=* *2,697)	Younger primiparas (*N *=* *978)	Older primiparas (*N *=* *477)	Younger multiparas (*N *=* *646)	Older multiparas (*N *=* *596)	Group comparisons and *p* values of Chi‐square tests
	*N* (%)	*N* (%)	*N* (%)	*N* (%)	*N* (%)	
Age, mean (*SD*)	33.0 (4.7)	29.6 (3.2)	37.7 (2.3)	30.5 (3.0)	37.6 (2.2)	
Marital status						<.001
Married	2,644 (98.0)	960 (98.2)	455 (95.4)	642 (99.4)	587 (98.5)	
Single	53 (2.0)	18 (1.8)	22 (4.6)	4 (0.6)	9 (1.5)	
Employment						<.001
Employed	1,251 (46.4)	493 (50.5)	267 (56.0)	226 (35.0)	265 (44.5)	
Not employed	1,445 (53.6)	484 (49.5)	210 (44.0)	420 (65.0)	331 (55.5)	
Missing	1 (0.0)	1 (0.1)	0 (0.0)	0 (0.0)	0 (0.0)	
Education						<.001
College or Graduate school	1,216 (45.1)	484 (49.5)	223 (46.8)	240 (37.2)	269 (45.1)	
Junior/high/vocational school or Junior college	1,478 (54.8)	493 (50.4)	254 (53.2)	406 (62.8)	325 (54.5)	
Missing	3 (0.1)	1 (0.1)	0 (0.0)	0 (0.0)	2 (0.3)	
Financial burden						<.001
Yes	1,223 (45.4)	447 (45.8)	179 (37.6)	345 (53.4)	252 (42.4)	
No	1,468 (54.6)	528 (54.2)	297 (62.4)	301 (46.6)	342 (57.6)	
Missing	6 (0.2)	3 (0.3)	1 (0.2)	0 (0.0)	2 (0.3)	
Type of delivery						<.001
Vaginal	2,241 (83.1)	868 (88.8)	367 (76.9)	536 (83.0)	470 (78.9)	
CS	455 (16.9)	110 (11.2)	110 (23.1)	110 (17.0)	125 (21.0)	
Missing	1 (0.0)	0 (0.0)	0 (0.0)	0 (0.0)	1 (0.2)	

CS, caesarean section; *SD*, standard deviation.

### 4.2 The course of maternal fatigue during the first 6 months postpartum

Our first aim was to identify the course of maternal fatigue during the first 6 months by focusing on maternal age and parity. Table 2 shows mean PAFS scores by group type at the five times. A mixed between/within‐subjects analysis of variance indicated statistically significant main effects of time [*F*(3.3, 8997.3) = 129.1, *p *<* *.001], indicating a significant difference between the five time points. The interaction effect between group type and time on mean PAFS scores was also significant [*F*(10.0, 8997.3) = 29.7, *p *<* *.001]. The simple main effects of time were significant for all groups: younger primiparas, *F*(4, 10820) = 2 35.7, *p *<* *.001; older primiparas, *F*(4, 10820) = 104.2, *p *<* *.001; younger multiparas, *F*(4, 10820) = 10.9, *p *<* *.001; and older multiparas, *F*(4, 10820) = 12.4, *p *<* *.001. Post hoc tests using the Bonferroni correction showed that mean PAFS scores for younger and older primiparas at 1 month postpartum significantly decreased to 2 and 4 months postpartum and then remained unchanged to 6 months postpartum. Mean PAFS scores for younger and older multiparas during hospital stay significantly increased to 1 month postpartum, then decreased to 2 months and again increased to 6 months postpartum. For older multiparas, a significant decrease on mean PAFS scores was further observed between two and 4 months postpartum.

**Table 2:** Means, standard deviations and pairwise multiple comparisons for Postnatal Accumulated Fatigue Scale scores by group and time (*N* = 2,697)

	Means (*SD*)					Pair Diffs. by time
	0 month	1 month	2 months	4 months	6 months	
YP (*n *=* *978)	9.18 (7.13)	9.35 (6.94)	6.80 (6.07)	6.24 (6.36)	6.49 (6.39)	†‡
OP (*n *=* *477)	9.61 (6.82)	10.09 (7.03)	7.81 (6.22)	6.85 (5.79)	7.10 (6.36)	†‡
YM (*n *=* *646)	6.29 (5.46)	8.11 (6.03)	6.94 (5.99)	6.73 (6.33)	7.40 (6.54)	*†§
OM (*n *=* *596)	7.16 (6.02)	9.00 (6.81)	7.90 (6.32)	7.31 (6.17)	7.94 (6.61)	*†‡§
Pair Diffs. by group	*†‡§	*‡	†||¶	†	*†	
Post hoc tests using the Bonferroni correction by time: *0 month different from 1 month, *p *<* *.05 †1 month different from 2 months, *p *<* *.05 ‡2 months different from 4 months, *p *<* *.05 §4 months different from 6 months, *p *<* *.05 Post hoc tests using the Bonferroni correction by group: *YP different from YM, *p *<* *.05 †YP different from OM, *p *<* *.05 ‡OP different from YM, *p *<* *.05 §OP different from OM, *p *<* *.05 ||YP different from OM, *p *<* *.05 ¶YM different from OM, *p *<* *.05						

*SD*, standard deviation; YP, Younger primiparas; OP, Older primiparas; YM, Younger multiparas; OM, Older multiparas.

**Figure 1:**
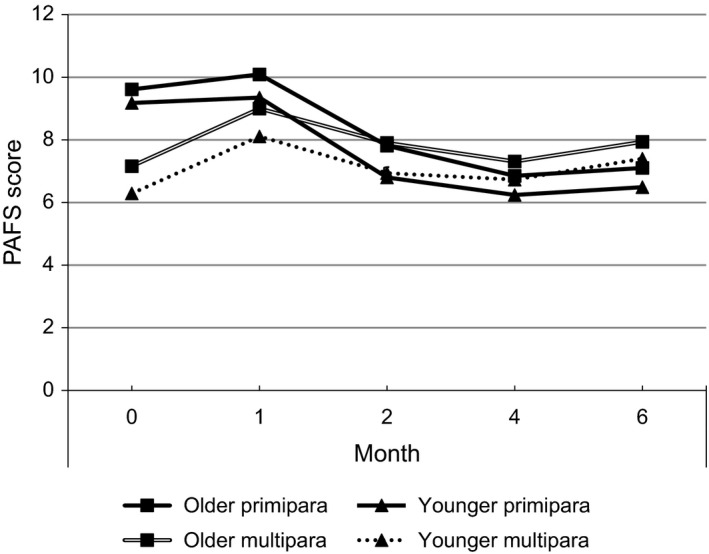
Trajectories of mean Postnatal Accumulated Fatigue Scale scores during the first 6 months postpartum (*N *=* *2,697)

The main effect for group type yielded an *F* ratio of *F*(3, 2693) = 5.1, *p *<* *.001, indicating a significant difference between four groups. The simple main effects of group type were significant at all time points: during hospital stay, *F*(3, 2693) = 38.8, *p *<* *.001; 1 month postpartum, *F*(3, 2693) = 8.6, *p *<* *.001; 2 months postpartum, *F*(3, 2693) = 5.9, *p *<* *.01; 4 months postpartum, *F*(3, 2693) = 3.9, *p *<* *.01; and 6 months postpartum, *F*(3, 2693) = 6.8, *p *<* *.001. Post hoc tests using the Bonferroni correction showed that younger and older primiparas had significantly higher mean PAFS scores than younger and older multiparas during hospital stay. At 1 month postpartum, younger and older primiparas had significantly higher mean PAFS scores than younger multiparas, but not older multiparas. At 2 months postpartum, older multiparas had significantly higher mean PAFS scores than younger and older primiparas and younger multiparas. At 4 months postpartum, older multiparas had significantly higher mean PAFS scores than younger primiparas. At 6 months postpartum, younger and older multiparas had significantly higher mean PAFS scores than younger primiparas. Trajectories of mean PAFS scores during the first 6 months postpartum are shown in Figure 1.

### 4.3 Factors associated with maternal fatigue during the first 6 months postpartum

Our second aim was to explore factors associated with maternal fatigue in two times: from hospital stay to 1 month postpartum and from 1 to 6 months postpartum. Table 3 presents the results of stepwise multiple regression in two times. Because our dependent variables were not just the total PAFS scores at one point in time, but a difference of degree of maternal fatigue at each time period (i.e. the total PAFS score at 1 month postpartum minus the total PAFS score during hospital stay and the total PAFS score at 6 months postpartum minus the total PAFS score at 1 month postpartum), associated factors were interpreted as contributing to variations of degree of maternal fatigue in each time period. That is, a positive value of β was interpreted as negatively contributing to the level of maternal fatigue. After adjusting for maternal age, type of delivery, parity and the initial level of maternal fatigue within each time period, common factors that contributed negatively to the level of maternal fatigue in the two times were: dissatisfaction with sleep, more concerns about child‐rearing, financial burdens, shorter meal times per day and dissatisfaction with emotional, informational and appraisal support.

**Table 3:** Stepwise multiple regression results for factors associated with PAFS scores in two time periods: from hospital stay to 1‐month postpartum; and from 1–6 months postpartum

Independent variables	From hospital stay to 1‐month postpartum		From 1‐ to 6‐month postpartum	
	β	P	β	P
Control variables				
Maternal age	−0.01	0.734	−0.02	0.274
Type of delivery (0 = vaginal, 1 = CS)	0.01	0.648	−0.01	0.615
Parity (0 = primiparity, 1 = multiparity)	0.05	0.005	0.12	<0.001
The PAFS score^b^	−0.60	<0.001	−0.66	<0.001
Explanatory variables				
Satisfaction with sleep^a^ (1 = yes, 2 = no)	0.25	<0.001	0.26	<0.001
Concerns about child‐rearing^b^	0.13	<0.001	0.09	<0.001
Satisfaction with emotional support^a^ (1 = yes, 2 = no)	0.07	0.001	0.06	0.004
Financial burden (0 = no, 1 = yes)	0.09	<0.001	0.06	<0.001
Meal times per day (min)^a^	−0.06	<0.001	−0.03	0.037
Satisfaction with informational support^a^ (1 = yes, 2 = no)	0.05	0.010	0.04	0.031
Satisfaction with appraisal support^a^ (1 = yes, 2 = no)	0.05	0.021	0.07	0.001
Duration between feeding and infant bedtime (min)^a^	0.07	<0.001		
Employment (0 = unemployed, 1 = employed)	−0.05	0.002		
Satisfaction with instrumental support^a^ (1 = yes, 2 = no)			0.07	0.001
Frequency of feeding during night^a^			0.06	<0.001
Hospitalization during pregnancy (0 = no, 1 = yes)			0.05	0.002
Marital status (0 = married, 1 = unmarried)			0.04	0.016
Adjusted *R* ^*2*^	0.34		0.41	
*F*	102.11	<0.001	129.37	<0.001

Higher values for meal times per day, frequency of feeding during night and concerns about child‐rearing respectively.

Control variables = type of delivery, maternal age, parity and the initial level of PAFS score.

CS, caesarean section; PAFS, Postnatal Accumulated Fatigue Scale.

a Data collected at 1‐month postpartum was used for analysis from hospital stay to 1 month postpartum and data at 6 months postpartum was used for analysis from 1 to 6 months postpartum.

b Data collected during hospital stay was used for analysis from hospital stay to 1 month postpartum and data at 1 month postpartum was used for analysis from 1 to 6 months postpartum.

Unique factors that contributed negatively to the level of maternal fatigue from hospital stay to 1 month postpartum were: longer duration between feeding and infant bedtime and employment. Unique factors that contributed negatively to the level of maternal fatigue from 1 to 6 months postpartum were: dissatisfaction with instrumental support, more frequent night time feeds, hospitalization during pregnancy and unmarried status.

## 5 DISCUSSION

### 5.1 The course of maternal fatigue during the first 6 months postpartum

Our findings showed one feature common to all groups of women regarding the course of maternal fatigue. That is, the level of maternal fatigue at 1 month postpartum was the highest and the severity of fatigue significantly decreased from 1–4 months postpartum. This trajectory is similar to that of depressive symptoms: the first month postpartum represented peak prevalence for depressive symptoms during the 6 months postpartum (Third‐person, Mori, Sakajo, Aoki et al., 2016). However, different features were also observed when focusing on maternal age and parity regarding the course of maternal fatigue. This was investigated by comparing four groups: younger primiparas, older primiparas, younger multiparas and older multiparas. Parity, not maternal age, was associated with the course of maternal fatigue during the first 6 months postpartum. Both younger and older primiparas showed similar trajectories: the PAFS scores were the highest at 1 month postpartum and they significantly decreased towards 2 and 4 months. Likewise, both younger and older multiparas showed similar trajectories: the PAFS scores during hospital stay significantly increased to 1 month postpartum and then decreased towards 2 or 4 months and then increased to 6 months postpartum.

Another important feature was that both younger and older primiparas showed significantly higher PAFS scores than younger and older multiparas during their hospital stays and at 1 month postpartum. However, no significant difference was observed between younger/older primiparas and older multiparas. This situation was reversed at later points in the study: both younger and older multiparas showed significantly higher PAFS scores than younger primiparas at 6 months postpartum. These findings indicate that primiparity may be a risk factor for maternal fatigue during the first month postpartum only and that multiparity becomes a risk factor for maternal fatigue at 6 months postpartum. These findings partly accord with our previous report on depressive symptoms: primiparity was a risk factor for depressive symptoms only during the first month postpartum and multiparity was not shown to be a risk factor in the later postpartum period (Third‐person, Mori, Sakajo, Aoki et al., 2016). This supports findings from previous studies where trajectories of fatigue and depressive symptoms overlap but remain distinct (Giallo, Gartland et al., 2015; Kuo et al., 2012).

In summary, our findings suggest a need for more intensive care in alleviating maternal fatigue for primiparas during the first month postpartum. Consideration should be given to postpartum duration and parity assuming primiparas and multiparas follow different courses of maternal fatigue during the first 6 months following birth.

### 5.2 Factors associated with maternal fatigue during the first 6 months postpartum

Our findings regarding the course of maternal fatigue during the first 6 months postpartum guided us to divide the 6 month period into two time periods: from hospital stay to 1 month postpartum and from 1–6 months postpartum. This method of analysis enabled us to examine factors associated with maternal fatigue at more appropriate times. Furthermore, our dependent variables were a difference of level of maternal fatigue in each time period, thus we were able to identify factors associated with variations of level of maternal fatigue within a designated time period. We also adjusted for the initial level of maternal fatigue within each period.

After adjusting for maternal age, type of delivery, parity and the initial level of maternal fatigue within each period, several associated factors were identified as common to both time periods: satisfaction with sleep, concerns about child‐rearing, satisfaction with social support, financial burdens and meal times per day.

#### 5.2.1 Satisfaction with sleep

Satisfaction with sleep was the strongest factor associated with maternal fatigue during the first 6 months postpartum. This finding accords with that from Australia, where the strongest predictor of maternal fatigue across the postpartum period from three to 7 months postpartum was poor sleep quality (Giallo, Seymour et al., 2015). Sleep plays an important role in good health throughout one's life, regardless of age, sex and significant life events such as childbirth. After childbirth, sleep disturbance typically occurs because of high‐need infant care. Frequent feeds and putting an infant to sleep at night result in poor sleep quality that includes: reduced minutes of total sleep time, frequent waking after sleep onset and poor sleep efficiency (Gay, Lee, & Lee, 2004).

Postpartum sleep quality was reported to have deteriorated the most during the first month postpartum and then to gradually improve to 4 months postpartum (Third‐person, Mori, Tsuchiya, Sakajo, Saeki et al., 2015). This time period accords with the finding in this study regarding the course of maternal fatigue: the first month represented the most severe fatigue and the level of fatigue significantly decreased from 1 to 4 months postpartum. We also previously reported that the first month postpartum represented peak prevalence for depressive symptoms (Third‐person, Mori, Sakajo, Aoki et al., 2016). Considering these findings together, sleep care should be provided more intensely during the first month postpartum in an attempt at alleviating maternal fatigue. This includes: educating mothers about altered sleep quality especially during the first month postpartum and providing information about how to manage sleep disturbance effectively.

Although many mothers are assumed to anticipate sleep disturbance after childbirth, they may not be aware of its profound impact on their health and may fail to make efforts to improve their sleep. Given that poor sleep quality can also be a sign of depression (American Psychiatric Association 2013), regular assessment of mothers’ satisfaction with sleep during the early postpartum period is important to provide appropriate care.

#### 5.2.2 Concerns about child‐rearing

‘Concerns about child‐rearing’ was the second strongest factor associated with maternal fatigue during the first 6 months postpartum. This factor consisted of concerns about newborn caretaking, one's own life and social support in our study. Mothers’ concerns about child‐rearing are closely related to their stress about child‐rearing or their perceptions of its difficulty. Our finding is partly consistent with those of previous studies, where mothers’ stress (Dunning & Giallo, 2012) or perceived difficulty in parenting (Kurth et al., 2011) were associated with maternal fatigue in the early postpartum period.

After childbirth, women need to acquire many new skills such as infant feeding, infant health management and adaptation to a new life with a new family system. This is considered part of the maternal adaptation process and it usually takes time (Mercer, 1995). We assumed that primiparas would lag behind in this aspect because of their inexperience in child‐rearing. Therefore, our finding was notable in that concerns about child‐rearing was consistently associated with maternal fatigue throughout the 6 months postpartum regardless of parity. This suggests that continued assessment of women's concerns about child‐rearing is important in alleviating maternal fatigue for all women.

An important consideration for multiparas regarding their concerns about child‐rearing should be noted. Combining the finding that maternal fatigue in both younger and older multiparas increased from four to 6 months postpartum, multiparas are assumed to have different concerns during this period. Taking care of not only a newborn but also older siblings may place an extra burden on multiparas, which becomes evident at later time points because of the maturational changes in the newborn. Nurses should be prudent enough to assume that primiparas and multiparas may well have some different concerns regarding child‐rearing.

#### 5.2.3 Satisfaction with social support

Satisfaction with social support, including emotional, informational and appraisal support, was consistently associated with maternal fatigue during the first 6 months postpartum. Social support generally plays an important role in one's health (Negron, Martin, Almog, Balbierz, & Howell, 2013). Specifically in the early postpartum period when infant care is demanding, social support has been reported to help prevent postpartum depressive symptoms (Third‐person, Mori, Tsuchiya et al., 2016), increase maternal confidence (Maehara, Mori, Tsuchiya, Iwata, Sakajo, Ozawa et al., 2016) and manage maternal fatigue (Cheng et al., 2015; Giallo et al., 2013).

Furthermore, the importance of social support can be emphasized when considering its close relationship with maternal sleep or concerns in child‐rearing. Both were found to be other factors associated with maternal fatigue in this study. Importantly, our findings showed the importance of women's perception of social support, which supports our previous report with the same sample. In that study, depressive symptoms were associated with women's satisfaction with social support, not with the quantity of the social support (Third‐person, Mori, Sakajo, Maehara et al., 2016). This suggests that women are not necessarily satisfied just because they receive plenty of support. Rather, health professionals should understand the existence of unwanted support: that is, receiving too much support is potentially damaging, especially when the support does not match the mother's desire or child‐rearing ethos (Maehara et al., 2014; Negron et al., 2013). Therefore, regular assessment of the mothers’ subjective experience of social support throughout the postpartum period will be important in alleviating maternal fatigue.

#### 5.2.4 Financial burden

Financial burden was the fourth most common factor associated with maternal fatigue during the first 6 months postpartum. This was similarly reported to be associated with maternal fatigue in a previous study (Giallo, Seymour et al., 2015). Financial burden or lower socio‐economic status has a potential impact on mothers’ childcare ability in that they may be forced to manage childcare with limited resources. This may include limited access to paid social support and having to eat poor meals, which may exacerbate maternal fatigue. Japanese mothers can be assumed to be hesitant to disclose financial problems to others, therefore, nurses can ask new mothers if they have financial concerns about childcare. This may create a good opportunity to discuss their financial burdens.

#### 5.2.5 Meal times per day

The last common factor associated with maternal fatigue was meal times per day: mothers who took a longer time for meals were more likely to have decreased levels of maternal fatigue. This is logical since adequate nutrition is important for good health. Surprisingly, however, we could find no study mentioning the importance of meals in relation to maternal fatigue during the postpartum period, though most nursing textbooks mention adequate food intake and good nutrition in maintaining health. It may be that most people take the importance of meals for granted. However, a qualitative study about postpartum maternal experiences indicated overworking as a characteristic of Japanese mothers. That is, they sometimes sacrifice their own time, including time for meals, to care for their children though they are indeed tired (Mori et al., 2014). Educating mothers about the importance of meals, assessing barriers that prevent mothers taking adequate meals and ensuring they are taking enough time for meals is another way maternal fatigue can be reduced.

#### 5.2.6 Other associated factors

Several factors were only found to be associated with maternal fatigue during specific time periods. Two performance factors included were: duration between feeding and infant bedtime from hospital stay to 1 month postpartum and frequency of night feeds from 1–6 months postpartum. Both of these factors may directly influence maternal fatigue, especially physical fatigue. The longer time needed to put an infant to sleep will deplete more of a mother's energy and this will be exacerbated during the first month postpartum when infants have irregular sleep patterns (Horiuchi, 1994). Likewise, frequent feeding during the night will disturb mothers’ sleep quality and this will continue to have a negative impact even when frequency of nocturnal feeding reduces towards 2 to 4 months postpartum (Third‐person, Mori, Tsuchiya, Sakajo, Saeki et al., 2015). Moreover, satisfaction with sleep and concerns about child‐rearing are considered to be closely related with both found to be associated with maternal fatigue in this study. This suggests that assessment of mothers’ feeding behaviours and concerns will be important in alleviating maternal fatigue.

Two situational factors included were employment and married status and one physiological factor was hospitalization during pregnancy. Mothers who had jobs were more likely to show a decrease in their level of fatigue during the first month postpartum. Those who were unmarried were more likely to have increased levels of fatigue from 1–6 months. These findings need further investigation; however, a possible explanation is that mothers with jobs may have more resilience than those without jobs. This may be more evident during the first month postpartum because new mothers are typically challenged with a dramatic change to their lifestyle during this period. Unmarried status could be discussed in relation to social support, because mothers who are not married might lack social support. Together with the finding that satisfaction with social support is an important factor associated with maternal fatigue, marital status should be included in assessing and planning care for alleviating maternal fatigue.

Mothers who experienced hospitalization during pregnancy were more likely to show increased levels of fatigue from 1–6 months postpartum. This suggests that these mothers are particularly vulnerable to fatigue, potentially brought about by maternal rest during hospitalization.

### 5.3 Study limitations

The first limitation of this study is that we only investigated the postpartum period and did not include any time before birth. Childbirth is a dramatic event that potentially has an impact on mothers’ physical and mental well‐being. Future research should investigate a longer period including before and after childbirth. This will help identify more appropriate times for interventions to support women in alleviating fatigue. The second limitation is that the variances in the study were 34% from hospital stay to 1 month postpartum and 41% from 1–6 months postpartum. This indicates that there were other significant factors associated with maternal fatigue during the first 6 months postpartum. The third limitation is that we only studied mothers who gave birth to a single child. The number of mothers giving birth to multiple children is increasing with the advancement of assisted reproductive technology in Japan (Saito et al., 2015). Mothers of multiple infants are assumed to be more fatigued because of caring for more babies at the same time. Therefore, generalization of our findings is limited to mothers who have given birth to a single child.

## 6 CONCLUSIONS

The course of maternal fatigue during the first 6 months postpartum showed both common and different features when focusing on maternal age and parity. A common feature in all groups of women was that the level of maternal fatigue at 1 month postpartum was the highest and the severity of fatigue significantly decreased from months 1–4 postpartum. A different feature was that parity, not maternal age, was associated with the course of maternal fatigue. Primiparas had the highest level of fatigue at 1 month postpartum and this significantly decreased towards 2 and 4 months postpartum, while the level of fatigue in multiparas significantly increased to 1 month postpartum and then decreased towards months two and four and then increased to 6 months postpartum. Another important different feature was that primiparas showed significantly higher levels of fatigue than multiparas during hospital stay and the level of fatigue was approximated by 1 month postpartum. This situation was reversed at later points in time: multiparas showed significantly higher levels of fatigue than younger primiparas at 6 months postpartum.

Regarding factors associated with varying levels of maternal fatigue within two designated periods, from hospital stay to 1 month postpartum and from 1–6 months postpartum, the following factors were identified as common to both time periods: satisfaction with sleep, concerns about child‐rearing, satisfaction with social support, financial burden and meal times per day. Unique factors from hospital stay to 1 month postpartum included: duration between feeding and infant bedtime and unemployment. Unique factors from 1–6 months postpartum included: frequency of feeding during the night, hospitalization during pregnancy and unmarried status.

## 7 RELEVANCE TO CLINICAL PRACTICE

There is a need for more intensive care in alleviating maternal fatigue for primiparas during the first month postpartum. Health professionals should pay attention to postpartum duration and parity assuming primiparas and multiparas follow different courses of maternal fatigue during the first 6 months following birth. Furthermore, the following factors should be assessed in alleviating maternal fatigue: satisfaction with sleep, concerns about child‐rearing, satisfaction with social support, financial burden and meal times per day.

## CONFLICT OF INTEREST

On behalf of all the authors, the corresponding author states that there is no conflict of interest.
